# The Sequence Ontology: a tool for the unification of genome annotations

**DOI:** 10.1186/gb-2005-6-5-r44

**Published:** 2005-04-29

**Authors:** Karen Eilbeck, Suzanna E Lewis, Christopher J Mungall, Mark Yandell, Lincoln Stein, Richard Durbin, Michael Ashburner

**Affiliations:** 1 Department of Molecular and Cellular Biology, Life Sciences Addition, University of California, Berkeley, CA 94729-3200, USA; 2 Howard Hughes Memorial Institute, Department of Molecular and Cellular Biology, Life Sciences Addition, University of California, Berkeley, CA 94729-3200, USA; 3 Cold Spring Harbor Laboratory, 1 Bungtown Road, Cold Spring Harbor, New York 11724, USA; 4 Sanger Institute, Wellcome Trust Genome Campus, Hinxton, Cambridgeshire, CB10 1SA, UK; 5 Department of Genetics, University of Cambridge, Downing Street, Cambridge, CB2 3EH, UK

## Abstract

The Sequence Ontology (SO) is a structured controlled vocabulary for the parts of a genomic annotation. SO provides a common set of terms and definitions that will facilitate the exchange, analysis and management of genomic data. Because SO treats part-whole relationships rigorously, data described with it can become substrates for automated reasoning, and instances of sequence features described by the SO can be subjected to a group of logical operations termed extensional mereology operators.

## Abstract 2

The goal of the Sequence Ontology (SO) project is to produce a structured controlled vocabulary with a common set of terms and definitions for parts of a genomic annotation, and to describe the relationships among them. Details of SO construction, design and use, particularly with regard to part-whole relationships are discussed and the practical utility of SO is demonstrated for a set of genome annotations from *Drosophila melanogaster*.

## Background

### Why a sequence ontology is needed

Genomic annotations are the focal point of sequencing, bioinformatics analysis, and molecular biology. They are the means by which we attach what we know about a genome to its sequence. Unfortunately, biological terminology is notoriously ambiguous; the same word is often used to describe more than one thing and there are many dialects. For example, does a coding sequence (CDS) contain the stop codon or is the stop codon part of the 3'-untranslated region (3' UTR)? There really is no right or wrong answer to such questions, but consistency is crucial when attempting to compare annotations from different sources, or even when comparing annotations performed by the same group over an extended period of time.

At present, GenBank [1] houses 220 viral genomes, 152 bacterial genomes, 20 eukaryotic genomes and 18 archeal genomes. Other centers such as The Institute for Genomic Research (TIGR) [2] and the Joint Genome Institute (JGI) [3] also maintain and distribute annotations, as do many model organism databases such as FlyBase [4], WormBase [5], The *Arabidopsis *Information Resource (TAIR) [6] and the *Saccharomyces *Genome Database (SGD) [7]. Each of these groups has their own databases and many use their own data model to describe their annotations. There is no single place at which all sets of genome annotations can be found, and several sets are informally mirrored in multiple locations, leading to location-specific version differences. This can make it hazardous to exchange, combine and compare annotation data. Clearly, if genomic annotations were always described using the same language, then comparative analysis of the wealth of information distributed by these institutions would be enormously simplified: Hence the Sequence Ontology (SO) project. SO began 2 years ago, when a group of scientists and developers from the model organism databases - FlyBase, WormBase, Ensembl, SGD and MGI - came together to collect and unify the terms they used in their sequence annotation.

The Goal of the SO is to provide a standardized set of terms and relationships with which to describe genomic annotations and provide the structure necessary for automated reasoning over their contents, thereby facilitating data exchange and comparative analyses of annotations. SO is a sister project to the Gene Ontology (GO) [8] and is part of the Open Biomedical Ontologies (OBO) project [9]. The scope of the SO project is the description of the features and properties of biological sequence. The features can be located in base coordinates, such as *gene *and *intron*, and the properties of these features describe an attribute of the feature; for example, a *gene *may be *maternally_imprinted*.

### SO terminology and format

Like other ontologies, SO consists of a controlled vocabulary of terms or concepts and a restricted set of relationships between those terms. While the concepts and relationships of the sequence ontology make it possible to describe precisely the features of a genomic annotation, discussions of them can lead to much lexical confusion, as some of the terms used by SO are also common words; thus we begin our description of SO with a discussion of its naming conventions, and adhere to these rules throughout this document.

Wherever possible, the terms used by SO to describe the parts of an annotation are those commonly used in the genomics community. In some cases, however, we have altered these terms in order to render them more computer-friendly so that users can create software classes and variables named after them. Thus, term names do not include spaces; instead, underscores are used to separate the words in phrases. Numbers are spelled out in full, for example *five_prime_UTR*, except in cases where the number is part of the accepted name. If the commonly used name begins with a number, such as 28S RNA, the stem is moved to the front - for example, *RNA_28S*. Symbols are spelled out in full where appropriate, for example, *prime*, *plus*, *minus*; as are Greek letters. Periods, points, slashes, hyphens, and brackets are not allowed. If there is a common abbreviation it is used as the term name, and case is always lower except when the term is an acronym, for example, *UTR *and *CDS*. Where there are differences in the accepted spelling between English and US usage, the US form is used.

Synonyms are used to record the variant term names that have the same meaning as the term. They are used to facilitate searching of the ontology. There is no limit to the number of synonyms a term can have, nor do they adhere to SO naming conventions. They are, however, still lowercase except when they are acronyms.

Throughout the remainder of this document, the terms from SO are highlighted in italics and the names of relationships between the terms are shown in bold. The terms are always depicted exactly as they appear in the ontology. The names of EM operators are underlined.

### SO, SOFA, and the feature table

To facilitate the use of SO for the markup of gene annotation data, a subset of terms from SO consisting of some of those terms that can be located onto sequence has been selected; this condensed version of SO is especially well suited for labeling the outputs of automated or semi-automated sequence annotation pipelines. This subset is known as the Sequence Ontology Feature Annotation, or SOFA.

SO, like GO, is an 'open source' ontology. New terms, definitions, and their location within the ontology are proposed, debated, and approved or rejected by an open group of individuals via a mailing list. SO is maintained in OBO format and the current version can be downloaded from the CVS repository of the SO website [10]. For development purposes, SOFA was stabilized and released (in May 2004) for at least 12 months to allow development of software and formats. SO is a directed acyclic graph (DAG), and can be viewed using the editor for OBO files, OBO-Edit [11].

The terms describing sequence features in SO and SOFA are richer than those of the Feature Table [12] of the three large genome databanks: GenBank [1], EMBL [13] and the DNA Data Bank of Japan (DDBJ) [14]. The Feature Table is a controlled vocabulary of terms describing sequence features and is used to describe the annotations distributed by these data banks. The Feature Table does provide a grouping of its terms for annotation purposes, based on the degree of specificity of the term. The relationships between the terms are not formalized; thus the interpretation of these relationships is left to the user to infer, and, more critically, must be hard-coded into software applications. Most of the terms in the Feature Table map directly to terms in SO, although the term names may have been changed to fit SO naming conventions. In general, SO contains a more extensive set of features for detailed annotation. There are currently 171 locatable sequence features in SOFA compared to 65 of the Feature Table. There are 11 terms in the Feature Table that are not included in SO. These terms fall into two categories: remarks and immunological features, both of which have been handled slightly differently in SO. A mapping between SO and the Feature Table is available from the SO website [10].

### Database schemas, file formats and SO

SO is not a database schema, nor is it a file format; it is an ontology. As such, SO transcends any particular database schema or file format. This means it can be used equally well as an external data-exchange format or internally as an integral component of a database.

The simplest way to use SO is to label data destined for redistribution with SO terms and to make sure that the data adhere to the SO definition of the data type. Accordingly, SO provides a human-readable definition for each term that concisely states its biological meaning. Usually the definitions are drawn from standard authoritative sources such as *The Molecular Biology of the Cell *[15], and each definition contains a reference to its source. Defining each term in such a way is important as it aids communication and minimizes confusion and disputes as to just what data should consist of. For example, the term *CDS *is defined as *a contiguous RNA sequence which begins with, and includes, a start codon and ends with, and includes, a stop codon*. According to SO, the sequence of a *three_prime_utr *does not contain the *stop_codon *- and files with such sequences are SO-compliant; files of *three_prime_utr *containing *stop_codons *are not. This is a trivial example, illustrating one of the simplest use cases, but it does demonstrate the power of SO to put an end to needless negotiations between parties as to the details of a data exchange. This aspect of SO is especially well suited for use with the generic feature format (GFF) [16]. Indeed, the latest version, GFF3, uses SO terms and definitions to standardize the feature type described in each row of a file and SO terms as optional attributes to a feature.

SO can also be employed in a much more sophisticated manner within a database. CHADO [17] is a modular relational database schema for integrating molecular and genetic data and is part of the Generic Model Organism Database project (GMOD) [18], currently used by both FlyBase and TIGR. The CHADO relational schema is extremely flexible, and is centered on genomic features and their relationships, both of which are described using SO terms. This use of SO ensures that software that queries, populates and exports data from different CHADO databases is interoperable, and thus greatly facilitates large-scale comparisons of even very complex genomics data.

Like GFF3, Chaos-XML [19] is a file format that uses SO to label and structure data, but it is more intimately tied to the CHADO project than is GFF3. Chaos-XML is a hierarchical XML mapping of the CHADO relational schema. Annotations are represented as an ontology-typed feature graph. The central concept of Chaos-XML is the sequence-feature, which is any sequence entity typed by SO. The features are interconnected via feature relationship elements, whereby each relationship connects a subject feature and an object feature. Features are located via featureloc elements which use interbase (zero-based) coordinates. Chaos-XML and CHADO are richer models than GFF3 in that feature_relationships are typed, and a more sophisticated location model is used. Chaos-XML is the substrate of a suite of programs called Comparative Genomics Library (CGL), pronounced 'seagull' [20], which we have used for the analyses presented in our Results section.

The basic types in SOFA, from which other types are defined, are *region *and *junction*, equivalent to the concepts of interiors and boundaries defined in the field of topological relationships [21]. A region is a length of sequence such as an *exon *or a *transposable_element*. A *junction *is the space between two bases, such as an *insertion_site*. Building on these basic data types, SOFA can be used to describe a wide range of sequence features. Raw sequence features such as assembly components are captured by terms like *contig *and *read*. Analysis features, defined by the results of sequence-analysis programs such as BLAST [22] are captured by terms such as *nucleotide_match*. Gene models can be defined on the sequence using terms like *gene*, *exon *and *CDS*. Variation in sequence is captured by subtypes of the term *sequence_variant*. These terms have multiple parentages with either region or junction. SOFA (and SO) can also be used to describe many other sequence features, for example, *repeat*, *reagent*, *remark*. Thus, SOFA together with GFF3 or Chaos-XML provide an easy means by which parties can describe, standardize, and document the data they distribute and exchange.

The SO and SOFA controlled vocabularies can be used for *de novo *annotation. Several groups including SGD and FlyBase now use either SO or SOFA terms in their annotation efforts. SO is not restricted to new annotations, however, and may be applied to existing annotations. For example, annotations from GenBank may be converted into SO-compliant formats using Bioperl [23] (see Materials and methods).

### SO relationships

**Figure 1:**
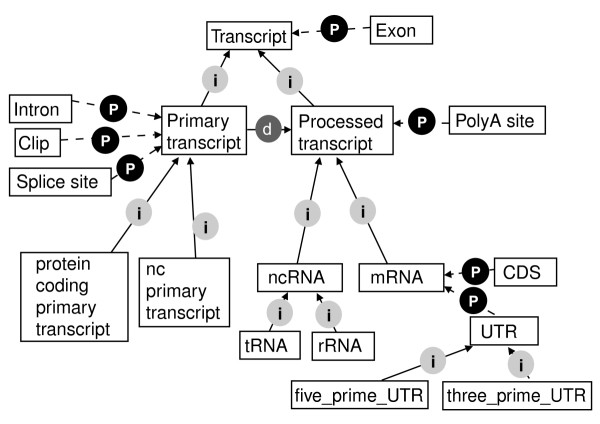
A section of the Sequence Ontology showing how terms and relationships are used together to describe knowledge about sequence. The **kind_of **relationships are depicted using arrows labeled with 'i', the **part_of **relationships use arrows with 'P' and the **derives_from **relationships with 'd'. By tracing the arrows that connect the terms, different logical inferences can be made regarding what a term 'is' and what are its allowable parts. For example, an *exon *is a **part_of **a *transcript*, a *tRNA *is a **kind_of ***ncRNA *which is a **kind_of ***processed_transcript*.

One essential difference between a controlled vocabulary, such as the Feature Table, and an ontology is that an ontology is not merely a collection of predefined terms that are used to describe data. Ontologies also formally specify the relationships between their terms. Labeling data with terms from an ontology makes the data a substrate for software capable of logical inference. The information necessary for making logical inferences about data resides in the class designations of the relationships that unite terms within SO. We detail this aspect of the ontology below. For purposes of reference, a section of SO illustrating the various relationships between some of its terms is shown in Figure 1.

Currently, SO uses three basic kinds of relationship between its terms: **kind_of**, **derives_from**, and **part_of**. These relationships are defined in the OBO relationship types ontology [24]. **kind_of **relationships specify what something 'is'. For example, an *mRNA *is a **kind_of ***transcript*. Likewise an *enhancer *is a **kind_of ***regulatory_region*. **kind_of **relationships are valid in only one direction. Hence, a *regulatory_region *is not a **kind_of ***enhancer*. One consequence of the directional nature of **kind_of **relationships is that their transitivity is hierarchical - inferences as to what something 'is' proceed from the leaves towards the root of the ontology. For example, an *mRNA *is a **kind_of ***processed_transcript *AND a *processed_transcript *is a **kind_of ***transcript*. Thus, an *mRNA *is a **kind_of ***transcript*. **kind_of **relationships are synonymous with **is_a **relationships. We adopted the '**kind_of**' notation to avoid the lexical confusion often encountered when describing relationships, as the phrase 'is a' is often used in conjunction with another relationships in English - for example 'is a part_of'.

SO uses the term **derives_from **to denote relationships of process between two terms. For example, an *EST ***derives_from **an *mRNA*. **derives_from **relationships imply an inverse relationship; **derives**. Note that although a *polypeptide ***derives_from **an *mRNA*, a *polypeptide *cannot be derived from an *ncRNA *(non-coding RNA), because no **derives_from **relationship unites these two terms in the ontology. This fact illustrates another important aspect of how SO handles relationships: children always inherit from parents but never from siblings. An *ncRNA *is a **kind_of ***transcript *as is an *mRNA*. Labeling something as a *transcript *implies that it could possibly produce a *polypeptide*; labeling that same entity with the more specific term *ncRNA *rules that possibility out. Thus, a file that contained ncRNAs and their polypeptides would be semantically invalid.

**part_of **relationships pertain to meronomies; that is to say 'part-whole' relationships. An *exon*, for example, is a **part_of **a *transcript*. **part_of **relationships are not valid in both directions. In other words, while an *exon *is a **part_of **a *transcript*, a *transcript *is not a **part_of **an *exon*. Instead, we say a *transcript ***has_part ***exon*. SO does not explicitly denote whole-part relationships, as every **part_of **relationship logically implies the inverse **has_part **relationship between the two terms.

Transitivity is a more complicated issue with regards to part-whole relationships than it is for the other relationships in SO. In general, **part_of **relationships are transitive - an *exon *is a **part_of **a *gene*, because an *exon *is a **part_of **a *transcript*, and a *transcript *is a **part_of **a *gene*. Not every chain of part-whole relationships, however, obeys the principle of transitivity. This is because parts can be combined to make wholes according to different organizing principles. Winston *et al*. [25] have described six different subclasses of the part-whole relationship, based on the following three properties: *configuration*, whether the parts have a structural or functional role with respect to one another or the whole they form; *substance*, whether the part is made of the same stuff as the whole (homomerous or heteromerous); and *invariance*, whether the part can be separated from the whole. These six relations and their associated **part_of **subclasses are detailed in Table 1.

Winston *et al*. [25] argue that there is transitivity across a series of **part_of **relationships only if they all belong to the same subclass. In other words, an *exon *can only be **part_of **a *gene*, if an *exon *is a **component_part_of **a *transcript*, and a *transcript *is **component_part_of **a *gene*. If, however, the two statements contain different types of **part_of **relationship, then transitivity does not hold.

By addressing the vague English term 'part of' in this way, Winston *et al. *solve many of the problems associated with reasoning across **part_of **relationships; thus, we are adopting their approach with SO. The parts contained in the sequence ontology are mostly of the type **component_part_of **such as *exon *is a **part_of ***transcript*, although there are a few occurrences of **member_part_of **such as *read *is a **part_of ***contig*.

### SO's relationships facilitate software design and bioinformatics research

Genomic annotations are substrates for a multitude of software applications. Annotations, for example, are rendered by graphical viewers, or, as another example, their features are searched and queried for purposes of data validation and genomics research. Using an ontology for sequence annotation purposes offers many advantages over the traditional Feature Table approach. Because controlled vocabularies do not specify the relationships that obtain between their terms, using the Feature Table has meant that relationships between features have had to be hard-coded in software applications themselves; consequently, adding a new term to the Feature Table and/or changing the details of the relationships that obtain between its terms has meant revising every software application that made use of the Feature Table. Ontologies mitigate this problem as all of the knowledge about terms and their relationships to one another is contained in the ontology, not the software.

SO-compliant software need only be provided with an updated version of the ontology, and everything else will follow automatically. This is because SO-compliant software need not hard-code the fact that a *tRNA *is a **kind_of ***transcript*; it need merely know that **kind_of **relationships are transitive and hierarchical and be capable of internally navigating the network of relationships specified by the ontology (see Figure 1) in order to logically infer this fact. This means that every time a new form of *ncRNA *is discovered, and added to SO, all SO-compliant software applications will automatically be able to infer that any data labeled with that new term is a **kind_of **transcript. This means that existing graphical viewers will render those data with the appropriate transcript glyph, and validation and query tools will automatically deal with this new data-type in a coherent fashion. Placing the biological knowledge in the ontology rather than in the software means that the ontology and the software that uses it can be developed, revised, and extended independently of one another. Thus ontologies offer the bioinformatics programming community significant opportunities as regards software design and the speed of the development cycle. Using an ontology does, however, mean that software applications must meet certain professional standards; namely, they must be capable of parsing an OBO file and navigating the network of relationships that constitute the ontology, but these are minimal hurdles.

SO facilitates bioinformatics research in ways that reach far beyond its utility as regards software design. For example, SO's **kind_of **relationships provide a subsumption hierarchy, or classification system for its terms. This added depth of knowledge greatly improves the searching and querying capabilities of software using SO. The ontology's higher-level terms may be used to query via inference, even if they are never used for annotation. We recommend that annotators label their data using terms corresponding to terminal nodes in the ontology. Transcripts, for example, might be annotated using terms such as **mRNA**, **tRNA**, and **rRNA **(see Figure 1). Note that doing so means that if, for example, non-coding RNA sequences are required for some subsequent analysis, then SO-compliant software tools can locate annotations labelled with the subtypes of ncRNA, and retrieve tRNAs and rRNAs to the exclusion of mRNAs, even though these data have not been explicitly labelled with the term **ncRNA**. Thus, many analyses become easy, for example, how many ncRNAs are annotated in *H. sapiens*? Of these what percent have more than one exon? Are any maternally imprinted? Moreover, using SO as part of a database schema ensures that such questions 'mean' the same thing in different databases.

SO also greatly facilitates the automatic validation of annotation data, as the relationships implied by an annotation can be compared to the allowable relationships specified in the ontology. For example, an annotation that asserts an *intron *to be **part_of **an *mRNA *would be invalid, as this relationship is not specified in the ontology (Figure 1). On the other hand, an annotation that asserted that an *UTR *sequence was **part_of ***mRNA *would be valid (Figure 1). This makes possible better quality control of annotation data, and makes it possible to check existing annotations for such errors when converting them to a SO-compliant format such as GFF3.

To summarize, by identifying the set of relationships between terms that are possible, we are also specifying the inferences that can be drawn from these relationships: that is, the software operations that can be carried out over the data. As a consequence, software is easier to maintain, SO can easily be extended to embrace new biological knowledge, quality controls can be readily implemented, and software to mine data can be written so as to be very flexible.

### EM operators and SO

SO also enables some modes of analyses of genomics data that are completely new to the field. One such class of analyses involves the use of extensional mereology (EM) operators to ask questions about gene parts. Although new to genomics, EM operators are well known in the field of ontology, where they provide a basis for asking and answering questions pertaining to how parts are distributed within and among different wholes (reviewed in [26,27]). These operators are usually applied to studies of how parts are shared between complex wholes - such as different models of automobiles or personal computers - for the purpose of optimizing manufacturing procedures. Below we explain how these same operators can be applied to the analyses of genomics data. Although these operators, difference and overlap, share the same name as topological operators, they are different as they function on the parts of an object, not on its geometric coordinate space. The topological operators, regarding the coincidence of edges and interiors - equality, overlap, disjointedness, containment and coverage of spatial analysis [21] - may also be applied to biological sequence.

EM is a formal theory of parts: it defines the properties of the **part_of **relationship and then provides a set of operations (Table 2) that can be applied to those parts. These operators are akin to those of set theory, but whereas set theory makes use of an object's **kind_of **relationships, EM operators function on an object's **part_of **relationships. Only wholes and their 'proper parts' are legitimate substrates for EM operations. Proper parts are those parts that satisfy three self-evident criteria: first, nothing is a proper part of itself (a proper part is part of but not identical to the individual or whole); second, if **A **is a proper part of **B **then the **B **is not a part of **A**; third, if **A **is a part of **B **and **B **is a part of **C **then **A **is a part of **C**.

Note that the third criterion of proper parts is that they obey the rule of transitivity. As we discussed earlier, not all **part_of **relationships are transitive. Accordingly, we have restricted our analyses (see Results and discussion) to component parts (Table 2).

**Figure 2:**
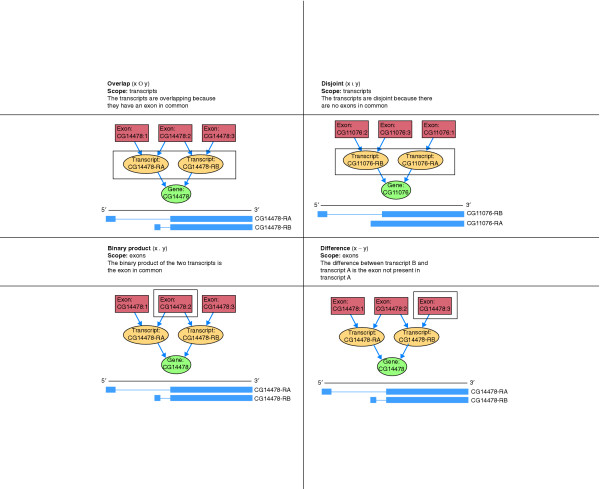
Using EM operations to characterize alternatively spliced transcripts and their exons. The EM operations overlap and disjoint can be used to characterize pair-wise relationships between alternative transcripts. Binary product and difference, on the other hand, pertain to exons shared, or not-shared between two alternative transcripts.

Figure 2 illustrates the effects of applying EM operations to analyze the relationships '*transcript *is a **part_of ***gene' *and '*exon *is a **part_of ***transcript*'. The EM operations overlap and disjoint pertain to relationships between transcripts, whereas difference and binary product pertain to exons. Two transcripts overlap if they share one or more exon in common. Two transcripts are disjoint if they do not share any exons in common. The exons shared between two overlapping transcripts are the binary product of the two transcripts, and the exons not shared in common comprise the difference between the two transcripts. The binary sum of two transcripts is simply the sum of their parts.

One key feature of EM operations is that they operate in 'identifier space' rather than 'coordinate space'. Two transcripts overlap only if they share a part in common rather than if their genomic coordinates overlap. Thus, two transcripts may be disjoint even if their exons partially overlap one another. This is one way in which EM analyses differ from standard bioinformatics analyses, and it has some interesting repercussions. This is particularly so with regard to modes of alternative splicing, as each of the EM operations suggests a distinct category by means of which two alternatively spliced transcripts can be related to one another. We further explore the potential of these operations to classify alternative transcripts and their exons below.

## Results and discussion

As part of a pilot project to evaluate the practical utility of SO as a tool for data management and analysis, we have used SO to name and enumerate the parts of every protein-coding annotation in the *D. melanogaster *genome. Doing so has allowed us to compare annotations with respect to their parts, for example, number of exons, amount of UTR sequence, and so on.

These data afford many potential analyses, but as our motivation was primarily to demonstrate the practical utility of SO as a tool for data management, rather than comparative genomics *per se*, we have focused more on what exon-transcript-gene part-whole relationships have to say about the annotations themselves, than what the annotations have to say about the biology of the genome. Accordingly, we have used EM-operators to characterize the annotations with respect to their parts, especially with regard to alternative splicing. The current version of FlyBase (5 August, 2004) contained 13,539 genes, (of which 10,653 have a single transcript and 2,886 are alternatively spliced), 18,735 transcripts and 61,853 exons.

### An EM-based scheme for classifying alternatively spliced genes

As we had characterized the parts of the annotations using SO, we were able to employ the EM operators over these parts. This proved to be a natural way to explore the relative complexity of alternative splicing, as the alternatively spliced transcripts have different combinations of parts: that is, exons. We grouped alternatively spliced transcripts into two classes. An alternatively spliced gene will contain overlapping transcripts if at least one of its exons is shared between two of its transcripts, and will have disjoint transcripts if one of its transcripts shares no exons in common with any other transcript of that gene. For the purposes of this analysis, we further classified disjoint transcripts as sequence-disjoint and parts-disjoint. We term two disjoint transcripts sequence-disjoint if none of their exons shares any sequence in common with one another; and parts-disjoint if one or more of their exons overlap on the chromosome but have different exon boundaries. Note that the three operations are pairwise, and thus not mutually exclusive. To see why this is, imagine a gene having three transcripts, A, B, and C. Obviously, transcript A can be disjoint with respect to B, but overlap with respect to C. Thus, we can speak of a gene as having both disjoint and overlapping transcripts.

**Figure 3:**
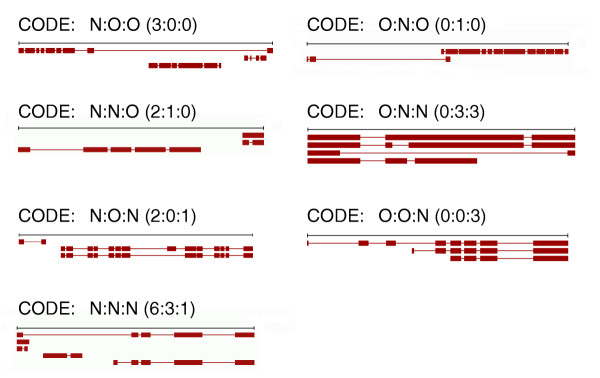
Examples of alternatively spliced genes from Entrez Gene at the NCBI. Of the seven classes of alternatively spliced genes, some classes are more likely to indicate annotation problems than others - particularly those genes having one or more sequence-disjoint transcripts. Parts-disjoint transcripts, on the other hand, are more suggestive of complex biology. Alternatively spliced genes having only overlapping transcripts (0:0:N) comprise the vast majority of instances.

The relative numbers of disjoint and overlapping transcripts in a genome says something about the relative complexity of alternative splicing in that genome. A gene may have any combination of these types of disjoint and overlapping transcripts, so we created a labeling system consisting of the seven possible combinations. We did this by asking three EM-based questions about the relationships between pairs of a gene's transcripts: How many pairs are there of sequence-disjoint transcripts? How many pairs are there of parts-disjoint transcripts? How many pairs are there of overlapping transcripts? Doing so allowed us to place that gene into one of seven classes with regards to the properties of its alternatively spliced transcripts. We also kept track of the number of times each of the three relationships held true for each pair combination. For example, a gene having two transcripts that are parts-disjoint with respect to one another would be labeled 0:1:0. Keeping track of the number of transcript pairs falling into each class provides an easy means to prioritize them for manual review. These results are summarized in Figure 3.

Of the alternatively spliced fly genes, none has a sequence-disjoint transcript, 275 have parts-disjoint transcripts, and 2,664 have overlapping transcripts, and 53 have both parts-disjoint and overlapping transcripts. The percentage of *D. melanogaster *genes in each category is shown in Table 3. Most alternatively spliced genes contain at least one pair of overlapping transcripts. These data also have something to say about the ways in which research and management issues are intertwined with one another with respect to genome annotation, as some aspects of these data are clearly attributable to annotation practice. The lack of any sequence-disjoint transcripts in *D. melanogaster*, for example, is due to annotation practice; in fact, current FlyBase annotation practices forbid their creation, the reason being that any evidence for such transcripts is evidence for a new gene [28]. This is not true for all genomic annotations. Annotations converted from the genomes division of GenBank to a SO-compliant form, were subjected to EM analysis, and inspection of the corresponding gene-centric annotations provided by Entrez Gene [29] revealed examples of genes that fall into each of the seven categories. Some of these annotations are shown in Figure 3.

The frequencies of genes that fall into each of the seven classes shown in Table 3 provides a concise summary of genome-wide trends in alternative splicing in the fly. This EM-based classification schema, when applied to many model organisms, from many original sources, makes very apparent the magnitude of the practical challenges that surround decentralized annotation, and the distribution and redistribution of annotations. Certainly, they highlight the need for data-management tools such as SO to assist the community in enforcing biological constraints and annotation standards. Only then will comparative genomic analyses show their full power.

### Exons as alternative parts of transcripts

EM-operators can also be used to classify the exons of alternatively spliced genes. Exons shared between two transcripts comprise the binary product of the two transcripts; whereas those exons present in only one of the transcripts constitute their difference (see Table 2 and Figure 2 for more information). These basic facts suggest a very simple, three-part classification system. If an exon is the difference between all other transcripts, then it is only in one transcript; we term these UNIQUE exons. If an exon is the difference of some transcripts, and the binary product of others, it is in a fraction of transcripts; we term these SOMETIMES_FOUND exons. And, if an exon is the binary product of all combinations of transcripts, then it must be in all transcripts; we term such exons ALWAYS_FOUND exons. Classifying exons in this way allows us to look more closely at alternative splicing from the exon's perspective.

As can be seen from Table 4, despite the low frequency of alternatively spliced genes, a large fraction of their exons are associated with alternatively spliced transcripts - almost 39%. A sizable proportion of SOMETIMES_FOUND and ALWAYS_FOUND exons are coding exons in some of the transcripts and entirely untranslated exons in others. In some cases, this is due to actual biology: some transcripts in *D. melanogaster *are known to produce more than one protein (see, for example [30]). In other cases, this situation appears to be a result of best attempts on the part of annotators to interpret ambiguous supporting evidence; in yet others the supporting data sometimes unambiguously points to patterns of alternative splicing that would seem to produce transcripts destined for nonsense-mediated decay [31]. Whatever the underlying cause, these exons, like the N:0:0 class annotations, should be subjected to further investigation.

**Figure 4:**
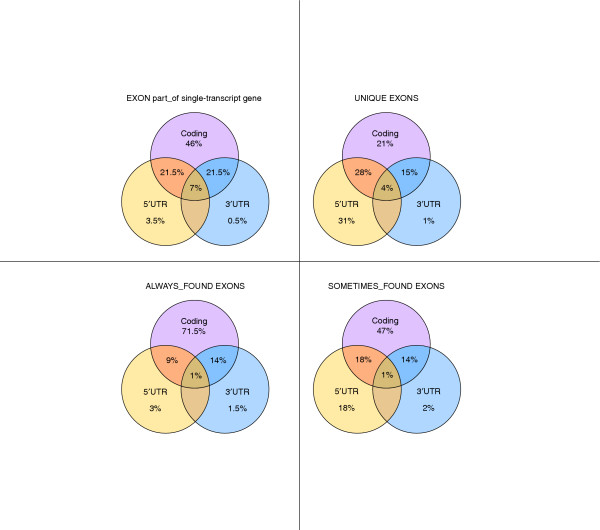
A series of Venn diagrams showing the relationship between exon class and coding potential. An exon may be fully protein coding, partially protein coding, or be fully UTR. An exon may be a **part_of **a single transcript gene (single-transcript genes), be a **part_of **either one (UNIQUE exons), all (ALWAYS_FOUND exons), or a fraction (SOMETIMES_FOUND exons) of transcripts in an alternatively transcribed gene.

To investigate these conclusions in more detail, we further examined each exon with respect to its EM-based class and its coding and untranslated portions. These results are shown Figure 4, and naturally extend the analyses presented in Table 4. First, regardless of exon class, most entirely untranslated exons are 5-prime exons; the lower frequency of 3-prime untranslated exons is perhaps due to nonsense-mediated decay [31], as the presence of splice junctions in a processed transcript downstream of its stop codon are believed to target that transcript for degradation. A second point made clear by the data in Table 4 is that alternatively spliced genes of *D. melanogaster *are highly enriched for 5-prime untranslated exons compared with single-transcript genes. Most of these exons belong to ALWAYS_FOUND; thus, there seems to be a strong tendency in *D. melanogaster *for alternative transcripts to begin with a unique 5' UTR region. This fact suggests that alternative transcription in the fly may, in many cases, be a consequence of alternative-promoter usage and perhaps tissue-specific transcription start sites. The high percentage of untranslated 5-prime UNIQUE exons in *D. melanogaster *may also be a consequence of the large numbers of 5' ESTs that have been sequenced in the fly [32].

Figure 4 also shows that most (> 95%) *D. melanogaster *ALWAYS_FOUND exons are coding. This makes sense, as it seems likely that one reason for an exon's inclusion in every one of a gene's alternative transcripts is that it encodes a portion of the protein essential for its function(s).

As with our previous analyses of alternative transcripts, our analyses of alternatively transcribed exons also illustrate the ways in which basic biology and annotation-management issues intersect one another. The fact that most ALWAYS_FOUND exons are entirely coding, for example, may have something important to say about which parts of a protein are essential for its function(s). Whereas the over-abundance of un-translated UNIQUE exons probably has more to say about the resources available to, and the protocols used by, the annotation project than it does about biology. Such considerations make it clear that the evidence used to produce an annotation is an essential part of the annotation. In this regard SO has much to offer, as it provides a rational means by which to manage annotation evidence in the context of gene-parts and the relations between those parts.

## Conclusion

We have sought to provide an introduction to the SO and justify why its use to unify genomic annotations is beneficial to the model organism community. We illustrate some of the ways in which SO can be used to analyze and manage annotations. Relationships are an essential component of SO, and understanding their role within the ontology is a basic prerequisite for using SO in an intelligent fashion. Much of this paper revolves around the **part_of **relationship because SO is largely a meronomy - a particular kind of ontology concerned with the relationships of parts to wholes. Extensional mereology (EM) is an area that is largely new to bioinformatics for which there are several excellent reference works available [26,27,33], and even a cursory examination of these texts will make it clear that EM has much to offer bioinformatics.

Using all of the relationships in SO allows us to automatically draw logical conclusions about data that has been labelled with SO terms and thereby provide useful insights into the underlying annotations. We have shown how SO, together with the EM-based operations it enables, can be used to standardize, analyze, and manage genome annotations.

Given any standardized set of genome annotations described with SO these annotations can then be rigorously characterized. For our pilot analyses, we focused on alternatively transcribed genes and their exons, and explored the potential of EM-operators to classify and characterize them. We believe that the results of these analyses support two principle conclusions. First, EM-based classification schemes are simple to implement, and second, they capture important trends in the data and provide a concise, natural, and meaningful overview of annotations in these genomes.

One criticism that might be justifiably leveled against the SO- and EM-based analyses presented here is that they are too formal, and that simpler approaches could have accomplished the same ends. As our discussion of **part_of **relationships made clear, however, reasoning across diverse types of parts is a complicated process; *ad-hoc *approaches will not suffice where the data are complex. The more formal approach afforded by SO means that analyses can be easily be extended beyond the domain of transcripts and exons to include many other gene parts and relationships as well - including evidence. It seems clear that over the next few years both the number and complexity of annotations will increase, especially with regard to the diversity of their parts. Drawing valid conclusions from comparisons of these annotations will prove challenging. That SO has much to offer such analyses is indisputable.

SO and SOFA provide the model organism community with a means to unify the semantics of sequence annotation. This facilitates communication within a group and between different model organism groups. Adopting SO terminology to type the features and properties of sequence will provide both the group and the community the advantages of a common vocabulary, to use for sharing and querying data and for automated reasoning over large amounts of sequence data.

## Materials and methods

SO and SOFA have been built and are maintained using the ontology-editing tool OBO-Edit. The ontologies are available at [34].

The FlyBase *D. melanogaster *[35] data was derived from the GadFly [36] relational database and converted to Chaos-XML using the Bio-chaos tools. The features were annotated to the deepest concept in the ontology possible, given the available information. For example, the degree of information in annotations was sufficiently deep to describe the transcript features with the type of RNA such as *mRNA*, or *tRNA*. It was therefore possible to restrict the analysis to given types of transcript. CGL tools were used to validate each of the annotations, iterate through the genes and query the features. EM-operators were applied to the part features of genes.

Other organism data was derived from the *genomes *section of GenBank [37]. GenBank flat files were converted to SO-compliant Chaos-XML using the script cx-genbank2chaos.pl (available from [19]) and BioPerl [23]. The BioPerl GenBank parser, Bio::SeqIO::genbank was used to convert GenBank flat files to Bioperl SeqFeature objects. Feature_relationships between these objects were inferred from location information using the Bioperl Bio::SeqFeature::Tools::Unflattener code. GenBank Feature Table types were converted to SO terms using the Bio::SeqFeature::Tools::TypeMapper class, which contains a hardcoded mapping for the subset of the GenBank Feature Table which is currently used in the *genomes *section of GenBank. The same Perl class was used to type the feature_relationships according to SO relationship types. The EM analysis was performed over the Chaos-XML annotations using the CGL suite of modules to iterate over the parts of each gene.

## Figures and Tables

**Table 1:** Six subclasses of part-whole relationships

Part_of subtype	Whole	Properties of relationship	Example
**component_part_of**	integral object	Functional/heteromerous/separable	A leg is a **part_of **a body. A regulatory_region is a **part_of **a *gene*.
**portion_part_of**	mass	Not functional/homomerous/separable	A slice is a **part_of **a cake. A restriction_fragment is **part_of **a chromosome.
**stuff_part_of**	object	Not functional/heteromerous/not separable	Carbon is a **part_of **a chromosome.
**member_part_of**	collection	Not functional/heteromerous/separable	A sheep is a **part_of **a flock. A *read *is a **part_of **a *contig*.
**place_part_of**	area	Not functional/homomerous/not separable	England is a **part_of **Britain.
**feature_part_of**	activity	Functional/heteromerous/not separable	Inhaling is a **part_of **breathing. Translation is **part_of **protein synthesis.

Column 1 gives the name of the subclass; column 2, the class or 'whole' to which such parts belong; column 3, the essential properties that define that particular part-whole relationship; and column 4 provides examples. Of the six classes only two - **component_part_of **and **member_part_of **occur in SO.

**Table 2:** The EM operators

EM operation	Definition
Overlap (x ○ y)	x and y overlap if they have a part in common.
Disjoint (x ι y)	x and y are disjoint if they share no parts in common.
Binary product (x . y)	The parts that x and y share in common.
Difference (x - y)	The largest portion of x which has no part in common with y.
Binary sum (x + y)	The set consisting of individuals x and y.

In each case x and y refer to two wholes. The first two operators are Boolean and pertain to whether two wholes share any parts in common; whereas the remainder return either the parts, or, in the case of binary sum, the wholes, that satisfy the operation.

**Table 3:** Percentage of each of the seven EM-based classes among the alternatively spliced genes in the *D. melanogaster *genome

Class	*D. melanogaster*
N:0:0	0%
N:N:0	0%
N:0:N	0%
N:N:N	0%
0:N:0	7.70%
0:N:N	1.83%
0:0:N	90.47%

The number of genes with one or more pairs of sequence-disjoint transcripts, no pairs of parts-disjoint transcripts, and no pairs of overlapping transcripts - denoted as N:0:0 - is given in the first row. Row 2 gives the number of genes having both sequence-disjoint and parts-disjoint transcripts, but no overlapping transcripts - these are N:N:0 genes. Rows 3 to 7 detail the counts for each of the remaining possible classes.

**Table 4:** Summary of the types of exons present in each of the genomes and their functions

	Exon part of gene with single transcript	Exon part of one transcript of alternatively spliced gene (UNIQUE)	Exon part of fraction of alternatively spliced transcripts (SOMETIMES_FOUND)	Exon part of all of the transcripts of alternatively spliced gene (ALWAYS_FOUND)
Percentage of all exons	60.1%	16.1%	5.2%	18.6%
Coding	94.5%	68%	73%	93%
Non-coding	4.5%	32%	19%	3.5%
Coding/non-coding	-	-	8%	3.5%

Exons of alternatively spliced genes were divided into three categories based on the binary product and difference operations. UNIQUE exons (column 2) occur in only a single transcript; SOMETIMES_FOUND exons (column 3) occur in some, but not all of a gene's alternatively spliced transcripts. ALWAYS_FOUND exons occur in every alternative transcript. The table rows show the breakdown of each exon class with respect to function, i.e., **coding exons **are those that consist at least partially of translated nucleotides, whereas **non-coding exons **consist entirely of UTR sequence. In some genes, an exon may be coding in one transcript and non-coding in another, depending on the annotated start and stop codons and the phase of the upstream intron; these exons are denoted as **coding/non-coding exons**. For reference purposes, the breakdown of exons in single-transcript genes is shown in column 1.
